# Understanding Kerion Celsi in Children: Diagnosis and Therapeutic Guidelines Through an Algorithm

**DOI:** 10.7759/cureus.58475

**Published:** 2024-04-17

**Authors:** Carla I Figueroa Basurto, Mario Shuchleib Cukiert, Eder R Juárez Durán, María E Vega-Memije, Ana L Ramírez Terán

**Affiliations:** 1 Department of Dermatology, "Dr. Manuel Gea González" General Hospital, Mexico City, MEX

**Keywords:** treatment algorithm, diagnostic algorithm, tinea capitis, scalp ringworm, kerion celsi

## Abstract

Kerion celsi (KC), known as scalp ringworm, is the most common dermatophytosis in children. In Mexico, it ranks fourth among dermatophytoses, with a frequency of 4%-10%. KC is the inflammatory variety of tinea capitis (TC), with the most common causative agents being *Microsporum canis* and *Trichophyton mentagrophytes. *We present the clinical case of a six-year-old male diagnosed with KC. Direct examination stained with chlorazol black and cultures were performed, yielding negative results. Histopathological study revealed spores and short hyphae within and surrounding the hair shaft. Treatment with itraconazole was initiated based on suspicion of *Microsporum *spp. from the trichoscopy findings. We propose a diagnostic and therapeutic algorithm for kerion celsi.

## Introduction

Kerion celsi (KC), known as scalp ringworm, is the most common dermatophytosis in children [1]. In Mexico, it ranks fourth among dermatophytoses, with a frequency of 4%-10%, making it a public health issue that predominates in rural and suburban areas [2]. KC is the inflammatory variety of tinea capitis (TC), with the most common causative agents being *Microsporum canis* and *Trichophyton mentagrophytes*. The clinical presentation is characterized by an inflammatory plaque that is tender to the touch and consists of multiple pustules, abscesses, ulcers, and honey-colored crusts [3]. It results from a hypersensitivity reaction to dermatophytes, leading to a severe inflammatory response with follicular pustules and neutrophilic infiltration around hair follicles, which may progress to granulomatous infiltration and resolve with scarring alopecia [4]. Trichoscopy is an easy and non-invasive method with higher sensitivity compared to direct examination (94% versus 49.1%) in diagnosing KC [5]. Its utility allows differentiation between *M. canis *and *T. mentagrophytes* [6]. We present a six-year-old male with a clinical, trichoscopic, and histopathological diagnosis of KC.

## Case presentation

**Figure 1:**
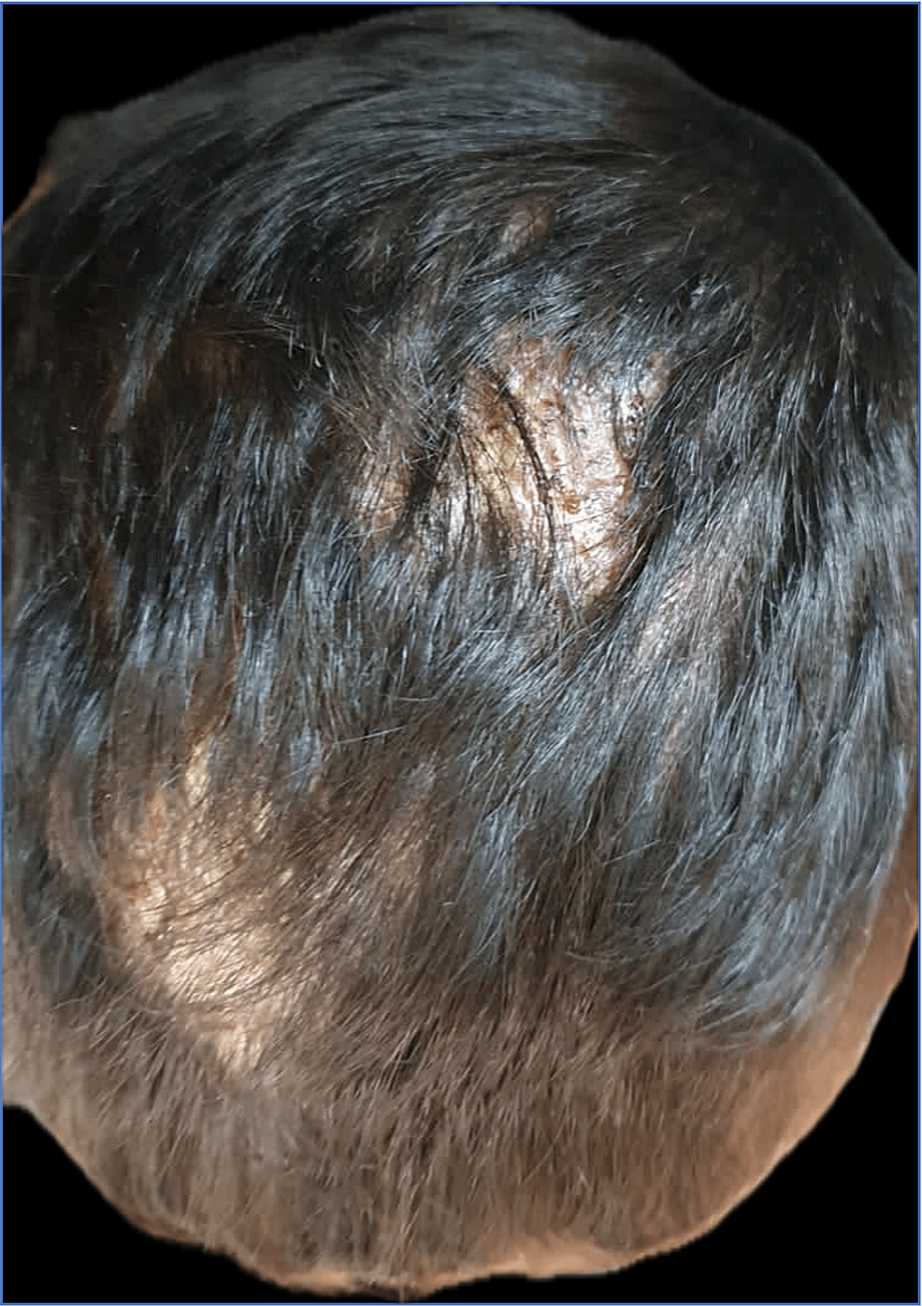
Clinical presentation Dermatosis on the scalp affecting the right parieto-frontal and left parieto-temporal regions.

**Figure 2:**
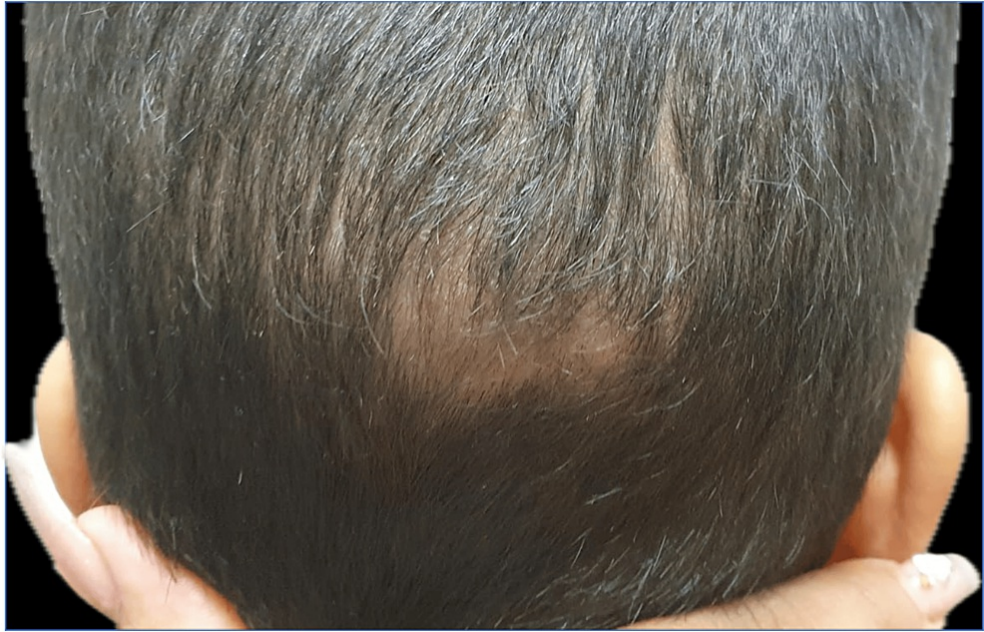
Clinical presentation Dermatosis on the scalp affecting the occipital region.

A six-year-old male from a rural area presented to the dermatology service with a localized dermatosis on the scalp that affected the right parieto-frontal, left parieto-temporal, and occipital regions. It was characterized by three plaques measuring 8 × 6 cm, 5 × 6 cm, and 6 × 4 cm, respectively, with regular borders. The first two had an erythematous base, pseudo-alopecia (affected hairs cut at the same level on the skin), pustules, and honey-colored and hematic crusts that fluctuated upon palpation and released purulent discharge (Figure 1). The third exhibited diffuse alopecia and fine scaling on the surface. Additionally, bilateral cervical lymphadenopathy was noted (Figure 2).

The patient's mother reported a progression of "pustules" over the past two months, with subsequent alopecia. The patient had previously received multiple topical azole treatments without improvement. The patient had prior diagnoses of Moebius syndrome, autism, and epilepsy, on risperidone for ongoing treatment.

**Figure 3:**
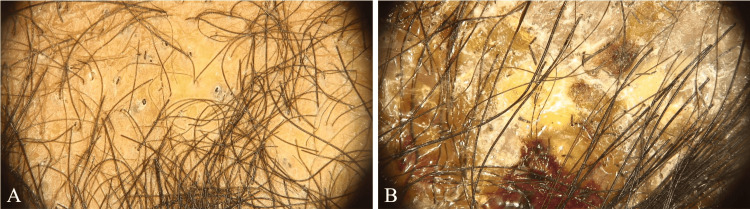
Trichoscopy A: Trichoscopy with zigzag hairs, broken hairs, black dots, and comma hairs. B: Trichoscopy with zigzag hairs, black dots, broken hairs, pustules, and interfollicular scaling.

**Figure 4:**
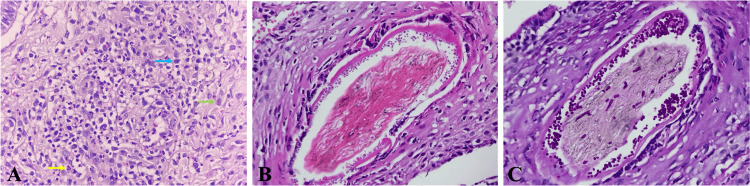
H&E and PAS staining A: Staining with H&E (40×) shows a fragment of the epidermis, with inflammatory infiltration of polymorphonuclear cells, neutrophils (yellow arrow), lymphocytes (blue arrow), and histiocytes (green arrow) in the dermis. B: Staining with H&E (60×) shows hyphae and spores around and inside the hair shaft. C: With PAS staining (60×), the hyphae and spores become more evident. H&E: hematoxylin and eosin, PAS: periodic acid-Schiff

We used the Fotofinder System GmbH® medicam 800HD (Columbia, MD), which revealed erythema, black dots, comma hairs, broken hairs, interfollicular scaling, honey-colored crusts, pustules, and pseudo-alopecia (Figure 3). Direct examination stained with chlorazol black yielded negative results. We observed agar growth over a period of four weeks on Mycosel®, which also yielded negative results. A biopsy was taken from the right parieto-temporal region and stained with hematoxylin and eosin (H&E). This biopsy showed ulcerated skin, crusts, colonies of coccoid bacteria, an epidermis with parakeratosis, acanthosis with pseudoepitheliomatous hyperplasia, spongiosis, and exocytosis of lymphocytes and neutrophils. In the dermis, there was a diffuse interstitial infiltrate of neutrophils, lymphocytes, and histiocytes with foreign body multinucleated giant cells, bare stems, and fragments of squamous epithelium immersed in the infiltrate. Periodic acid-Schiff (PAS) staining revealed short spores and hyphae within and surrounding the hair shaft (Figure 4), which was type 4 (suppurative folliculitis (SF) with granulomatous and suppurative dermatitis (SD)) according to Arenas et al. [4].

Based on the clinical, trichoscopic, and histopathological findings, a diagnosis of KC was established. Liver function tests resulted in no abnormalities; therefore, treatment was initiated with itraconazole 6 mg/kg/day for four weeks and prednisone 0.5 mg/kg/day for seven days. The patient experienced complete remission of the dermatosis and resolution of the scarring alopecia.

## Discussion

Literature review

Definition

Tinea capitis has different clinical presentations, subdivided into non-inflammatory and inflammatory types. KC is among the inflammatory presentations of tinea capitis, also known as scalp ringworm [7]. It is considered a superficial mycosis caused by dermatophytes, parasitic fungi of keratin, especially of the genera* Trichophyton *spp.*, Microsporum *spp., and* Epidermophyton *spp. [3].

Epidemiology

In Mexico, tinea capitis ranks fourth among dermatophytosis, making it a public health issue that predominates in rural and suburban areas with a frequency of 4%-10%. It is caused by *M. canis *in 80% of the population and *T. tonsurans* in 15%. It affects both sexes equally and involute at puberty [2,8]. Scalp ringworm is observed in the majority of cases in children, up to 98%, between the ages of three and seven years old, and it is considered the most common cutaneous mycosis in this population [7].

Bonifaz et al. [9]conducted a clinical mycological study of 125 cases involving children between 0 and 15 years with an average age of 7.2 years. The study shows that the predominant gender was male, with 67 (53.6%) cases, and 58 (46.6%) were female. The most frequent age of presentation for tinea capitis was 6-10 years, with 79 patients in the study. Martínez-Suárez et al. [10]also conducted a retrospective study with 122 cases from two dermatology services in Mexico City. The average age was 6.1 years, with a predominance of female gender, with 71 (58.1%) patients [10].

Etiology

Anthropophilic species associated worldwide with KC include *T. tonsurans* (the most common fungal species in pediatric KC), *T. violaceum*, and *T. soudanense*. Zoophilic species are *T. mentagrophytes*, *T. verrucosum*, *T. benhamiae*, and *M. canis*. *Nannizzia gypsea* (formerly *M. gypseum*) is a geophilic species identified occasionally in KC [1].

In Mexico, the most common species in KC is *M. canis *[9,10]. In the first study, a predominance of *M. canis *was obtained at 11.6%, followed by *T. tonsurans *(16.8%) [9]. In the second study, *M. canis *was isolated in 75 (61.5%) cases, while the second etiological agent was *T. tonsurans* isolated in 36 (29.5%) patients [10]. Therefore, it continues to be demonstrated that *M. canis *is the predominant etiological agent in Mexico.

Clinical presentation

KC is characterized by a painful inflammatory mass upon palpation, occasionally accompanied by lymphadenopathy; the lymph nodes most involved are the posterior cervical and posterior auricular nodes [11]. It is typically a single and limited lesion, but it can also be giant and multiple. KC begins as a dry ringworm composed of one or several pseudo-alopecic plaques, with scaling and short hairs. Subsequently, erythema and inflammation appear, leading to a well-defined, painful lesion covered with numerous pustules, and honey-colored crusts. The most significant symptom of this type of scalp ringworm is pain. If the process continues, short hairs are gradually expelled or remain under the inflammatory process. In approximately eight weeks, tissue response and, above all, cellular immunity eliminate the parasite, but this leaves areas of scarring alopecia with fibrosis as a consequence, as the hair follicle is constantly attacked [1,3,12].

Diagnosis

**Figure 5:**
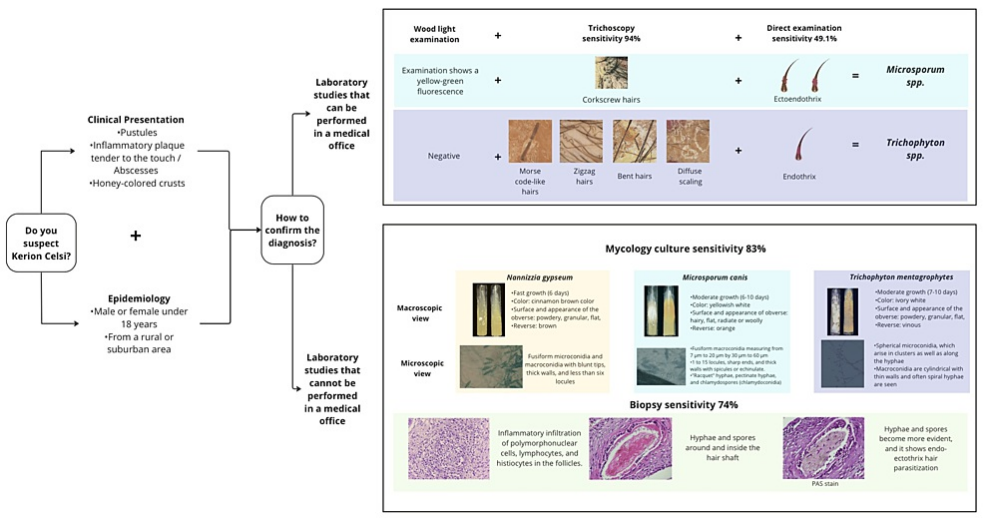
Diagnostic algorithm for kerion celsi Note: Author's own creation

The diagnosis of kerion celsi is based on clinical examination and laboratory tests. It includes the patient's history (disease course, other affected individuals among the patient's social contacts, exposure to animals, and trips), inspection (other clinical manifestations of dermatophytosis such as tinea corporis or onychomycosis), and the use of trichoscopy or Wood light (365 nm). Laboratory tests primarily consist of direct examination stained with chlorazol black and culture. In case of suspecting any other differential diagnosis, it is suggested to take a biopsy of the lesion for histological examination (PAS staining) (Figure 5) [13].

Wood Light Examination

The diagnosis of TC caused by *Microsporum* spp. may be considered to be confirmed if the Wood light examination shows a yellow-green fluorescence. However, the sensitivity of this test for fungal scalp infections is not high enough to definitively rule out a *Microsporum* infection merely based on the absence of a yellow-green fluorescence [12].

Direct Examination Stained With Chlorazol Black

Samples for direct examination are obtained by scraping with a slide or a surgical blade; in the case of scalp ringworm, some hairs can be extracted with tweezers. Visualization is performed with potassium hydroxide, with or without dimethyl sulfoxide, or with chlorazol black, which facilitates the observation of fungal elements inside or around the hair, as well as with calcofluor white and fluorescence microscopy. Under the microscope, hairs can present five types of parasitism, two endothrix and three ectoendothrix. Endothrix parasitism is distinguished by trichophytic parasitism (*T. tonsurans) *and favic parasitism (*T. schoenleinii*). On the other hand, ectoendothrix parasitism is distinguished by microsporic parasitism (*M. canis)*, megasporated parasitism (*T. verrucosum*), and microide parasitism (*T. mentagrophytes*) [14].

Culture

The culture is performed on standard media with or without antibiotics. Fungi tolerate cycloheximide and alkalinize the medium when grown on glucose or peptone agar. To stimulate fruiting, potato agar or corn meal agar is used [14].

Histopathology

The histological study is useful in patients with negative cultures, it reveals spores surrounding the hair follicle and hyphae within the follicle [13]. H&E slides were categorized according to five inflammatory patterns: perifolliculitis (PF), suppurative folliculitis (SF), SF with suppurative dermatitis (SD), SF with suppurative and granulomatous dermatitis (SGD), and SGD with fibrosing dermatitis (FD) [4].

Treatment

**Figure 6:**
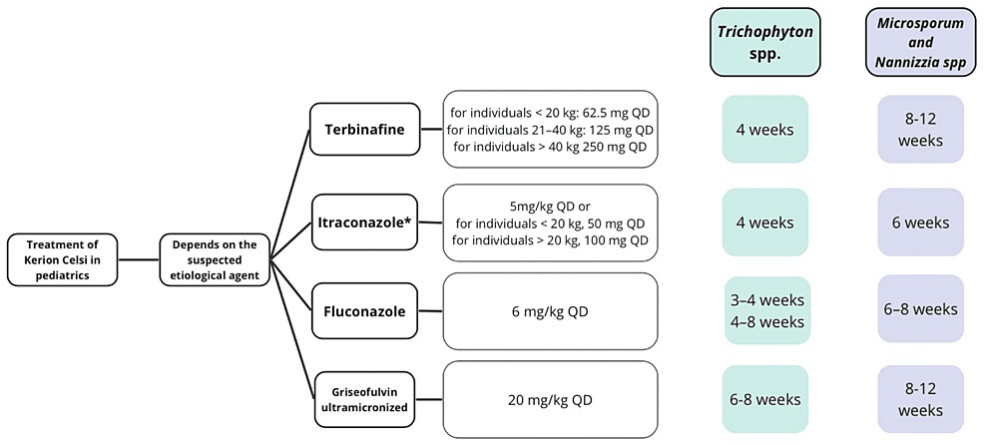
Treatment algorithm for kerion celsi *Taken with the main meal, suspension (fasting, no food intake for one hour) [12] QD: quaque die (once a day) Modified from Mayser P, Nenoff P, Reinel D, et al.: S1 guidelines: tinea capitis. J Dtsch Dermatol Ges. 2020, 18:161-79. 10.1111/ddg.14026 [12]

Treatment aims to cure and prevent cicatricial alopecia. Oral antifungals are used for this purpose, and the choice depends on the suspected etiological agent (Figure 6) [12].

## Conclusions

The diagnosis of KC is based on clinical suspicion and physical examination. We propose an algorithm based on what is already described in the literature so that physicians from different specialties who are not dermatologists can diagnose this condition in a medical office, following our algorithm proposal, and also become acquainted with the various diagnostic options available for KC, as well as its treatment. We emphasize the importance of an early diagnosis of KC to avoid sequelae such as scarring alopecia in pediatric patients.
